# Plasma Galectin-4 and Charcot-Leyden Crystal Protein/Galectin-10 as Emerging Biomarkers of Metabolically Induced Inflammation in Patients with Psoriasis

**DOI:** 10.3390/ijms262110339

**Published:** 2025-10-23

**Authors:** Anna Baran, Julia Nowowiejska, Julia Parzych, Justyna Magdalena Hermanowicz, Beata Sieklucka, Dariusz Pawlak, Iwona Flisiak

**Affiliations:** 1 Department of Dermatology and Venereology, Medical University of Bialystok, Zurawia 14 St., 15-540 Bialystok, Poland; anna.baran@umb.edu.pl (A.B.); julia.nowowiejska@umb.edu.pl (J.N.); iwona.flisiak@umb.edu.pl (I.F.); 2 Department of Pharmacodynamics, Medical University of Bialystok, Mickiewicza 2C St., 15-089 Bialystok, Poland; justyna.hermanowicz@umb.edu.pl (J.M.H.); beata.sieklucka@umb.edu.pl (B.S.); dariusz.pawlak@umb.edu.pl (D.P.)

**Keywords:** galectin 4, galectin 10, Charcot-Leyden crystal protein, atherosclerosis, psoriasis, comorbidities, metabolic syndrome, systemic therapy

## Abstract

Psoriasis is a complex inflammatory disease related to cardiometabolic disorders (CMDs). Galectin-4 (gal-4) is involved in biological processes such as lipid raft stabilization, intestinal inflammation and tumorigenesis. Charcot-Leyden crystals (CLCs), inter alia, Charcot-Leyden crystal/galectin-10 (CLC/gal-10), are involved in eosinophil-derived diseases. To date, neither of these galectins has been investigated in the context of psoriasis. The study aimed to evaluate serum galectin-4 and -10 levels in psoriatic patients and explore potential relationships with disease activity, metabolic or inflammatory indices. Blood samples were collected from 60 patients with plaque-type psoriasis and 30 healthy volunteers and evaluated using an Enzyme-Linked Immunosorbent Assay (ELISA). Morphological and biochemical indices were measured using routine laboratory techniques. Plasma gal-4 and gal-10 concentrations were significantly higher in patients with psoriasis than in the control group (*p* < 0.05). Galectins did not correlate with the Psoriasis Area Severity Index (PASI) nor age (*p* > 0.5); however, gal-4 showed a significant positive correlation with Body Mass Index (BMI), psoriasis duration (*p* = 0.03), and transaminase activity. Both proteins were the highest in obese psoriatics (*p* < 0.05). The results indicate that galectin-4 and galectin-10 may be involved in the pathophysiological mechanisms underlying CMDs in psoriatics. These galectins represent promising candidates for biomarkers of metabolically driven inflammation, with galectin-4, in particular, emerging as a potential indicator of hepatic dysfunction in psoriatic patients.

## 1. Introduction

Psoriasis is a long-term systemic inflammatory disorder that can result in both skin-related and systemic complications [1]. It can present with a variety of multisystemic manifestations, such as psoriatic arthritis, obesity, diabetes mellitus (DM), cardiovascular diseases (CVD), malignancies, chronic kidney disease (CKD), psychiatric conditions, inflammatory bowel disease (IBD), non-alcoholic fatty liver disease (NAFLD), all of which are associated with persistent inflammation [2,3,4]. The prevalence of metabolic syndrome (MS) in psoriasis patients ranges from 20% to 50% and increases with disease severity, with factors such as elevated triglycerides, high blood pressure and impaired glucose metabolism contributing to the increased cardiovascular risk [2]. Psoriasis is an independent risk factor for mortality and is associated with an elevated risk of cardiovascular comorbidities [5,6]. The complex interplay between psoriasis and various comorbidities can be attributed to shared genetic and immunological mechanisms, as well as oxidative stress and inflammation driven by systemic metabolic factors.

Galectins are β-galactoside-binding lectins with one or two carbohydrate recognition domains (CRDs) of about 130 amino acids. Based on their structure, galectins are classified into three types: prototypic, chimeric and tandem-repeat. Prototypic galectins feature a single carbohydrate recognition domain (CRD), while tandem-repeat galectins are made up of two homologous CRDs linked by a peptide linker. Chimeric galectins contain one CRD at the C-terminal end and a non-lectin tail at the N-terminal end that facilitates oligomerization [7,8]. At least 12 galectins have been identified in humans and at least 15 members of this family have been described in mammals [9,10]. They are present in various cellular locations, including the nucleus, cytosol, organelles or extracellular environment [8]. Galectins play a role in various intracellular and extracellular pathways that contribute to the regulation of metabolism and inflammation [9]. Interestingly, they may exert opposing effects, serving as negative or positive modulators of the same processes [9,10,11]. These molecules have not yet been extensively investigated in the context of psoriasis; therefore, they have become the focus of our research interest. Our previous work revealed that galectin-3 can influence the course of psoriasis and cardiometabolic comorbidities; moreover, galectins-1, 2 and 12 may be involved in metabolic complications associated with psoriasis [11,12]. Given their potential significance and the current paucity of data, we decided to further investigate the possible involvement of galectins in the pathogenesis of psoriasis.

Galectin-4 is a tandem-repeat galectin that contains two domains connected by a linker region [8,13]. Galectin-4 is encoded by the LGALS4 gene, located on chromosome 19q13.2, and is primarily expressed in the gastrointestinal tract [8,14,15,16]. Among the ligands of galectin-4, human blood group antigens, glycoproteins, mucin-like membrane MUC1, glycosphingolipids, sulfated cholesterol or sulfates can be distinguished [10]. The protein serves various functions in cell adhesion, intestinal inflammation, wound healing, tumor progression, lipid raft stabilization, protein apical trafficking, and axon growth. It has been shown that galectin-4 directly stimulates CD4+ T cells to produce IL-6, which contributes to the development of IBD, a condition closely related to psoriasis. Galectin-4 in serum has the potential to be a predictor of colon and breast cancer and is engaged in the peritoneal metastasis of malignant gastric cancer [10,14]. Moreover, recent studies have linked circulating gal-4 to obesity, diabetes and cardiovascular disease, which are crucial comorbidities of psoriasis. Pichler et al. reported that gal-4 drives proinflammatory NF-κB signaling and matrix metalloproteinase activation in human chondrocytes, demonstrating its ability to regulate tissue remodeling beyond the gut [17]. Additionally, a higher plasma level of galectin-4 is associated with an increased likelihood of forming carotid plaque, which is commonly observed in patients with psoriasis [18]. To date, the protein has not been studied in the context of psoriasis. Therefore, the broad biological roles and extensive involvement of gal-4 in various pathological processes and diseases significantly associated with psoriasis have sparked our interest and motivated us to explore this subject further.

Galectin-10, also known as Charcot-Leyden crystal (CLC) protein, is a prototypic protein that forms dimers and is encoded by the CLC gene located on chromosome 19q13.2 [19]. Eosinophils, basophils and macrophages contain numerous galectin-10 proteins [20,21]. That indicates the presence of the protein in type 2 immune responses. Protein composes Charcot-Leyden crystals, which accumulate in the tissues and are linked with allergic diseases, parasitic infections, hematological malignancies or even solid tumors, like melanoma or gastric cancer [22]. It was found that atopic dermatitis (AD) is associated with an increased amount of galectin-10, resulting from the overexpression of this protein by CD3+ T cells and IL-22-producing CD4+ T cells [22,23]. Psoriasis, along with AD, is an inflammatory skin disease, which motivates to search for a connection between galectin-10 and psoriasis.

The promising yet unexplored effects and potential applications of the two galectins prompted us to investigate them in the plasma of psoriatic patients, as well as their association with disease severity, indices of metabolic and inflammatory status and the risk of related complications.

## 2. Results

Baseline characteristics of the study and the volunteer group are presented in Table 1.

### 2.1. Galectin-4

**Figure 1:**
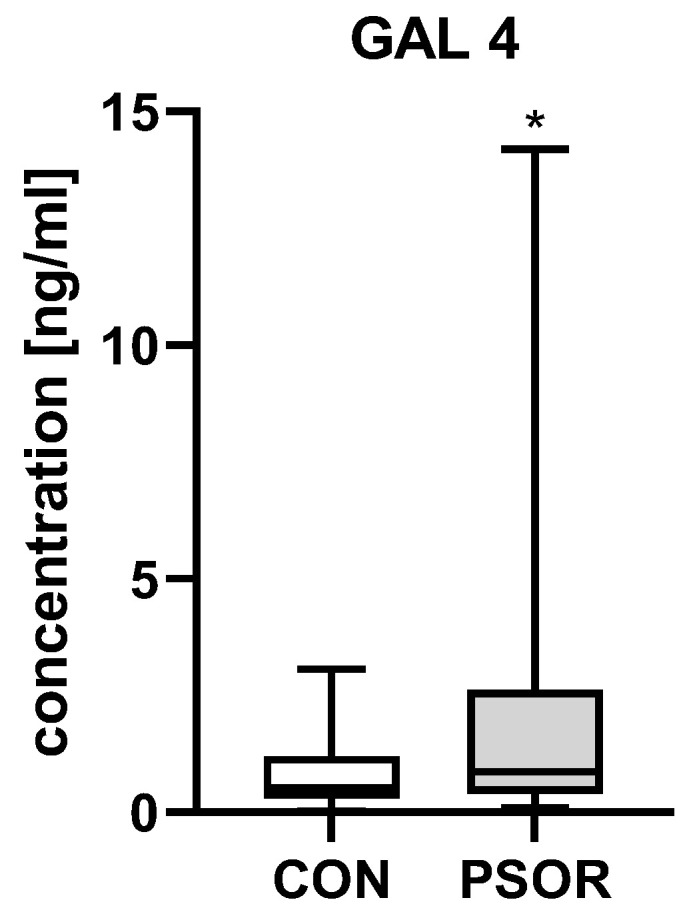
Plasma gal-4 concentration in patients compared to controls. * means a statistical significance with *p* < 0.05. (CON *n* = 30, PSOR *n* = 60).

Plasma gal-4 concentration was significantly higher in patients than in controls (*p* < 0.05) (Figure 1).

**Figure 2:**
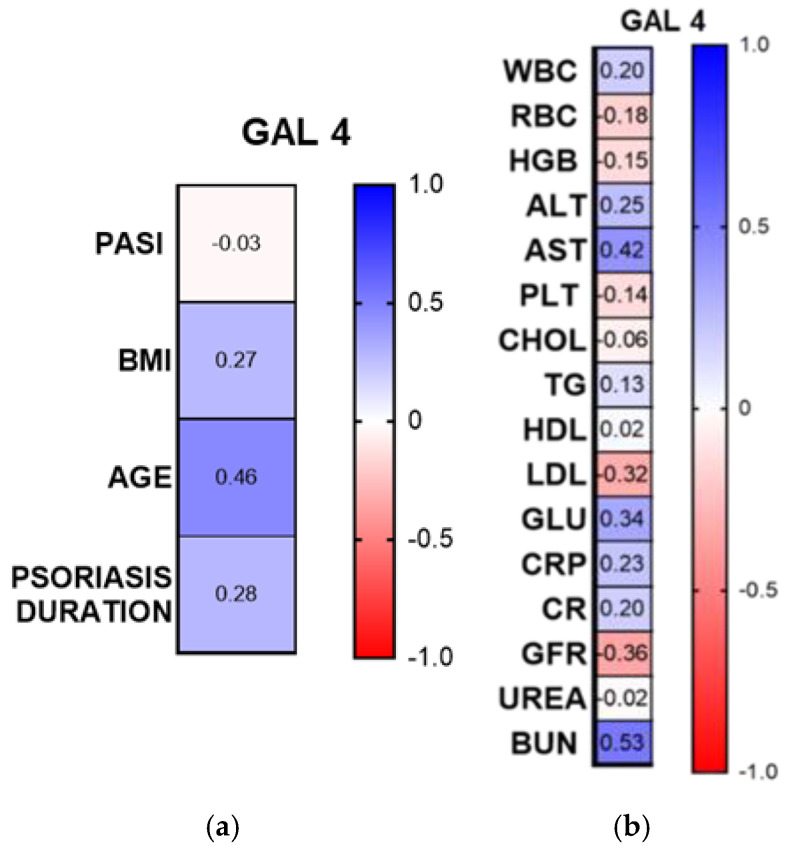
Correlations between plasma galectin-4 concentrations and clinical parameters (**a**) or laboratory indices (**b**). TGs, triglycerides; ALT, alanine aminotransferase; AST, asparagine aminotransferase; GLU, fasting glucose; Chol, total cholesterol; HDL, high-density lipoprotein; LDL, low-density lipoprotein; RBC, red blood cells; WBC, white blood cells; PLT, platelets; HGB, hemoglobin; CR, creatinine. *n* = 60.

Galectin-4 did not correlate with psoriasis severity evaluated with PASI score nor age (R = −0.03, *p* = 0.80; R = 0.46, *p* = 2.10, respectively) (Figure 2a). However, galectin-4 was significantly positively related to BMI and psoriasis duration (R = 0.27, *p* = 0.03; R = 0.28, *p* = 0.03, respectively) (Figure 2a).

**Figure 3:**
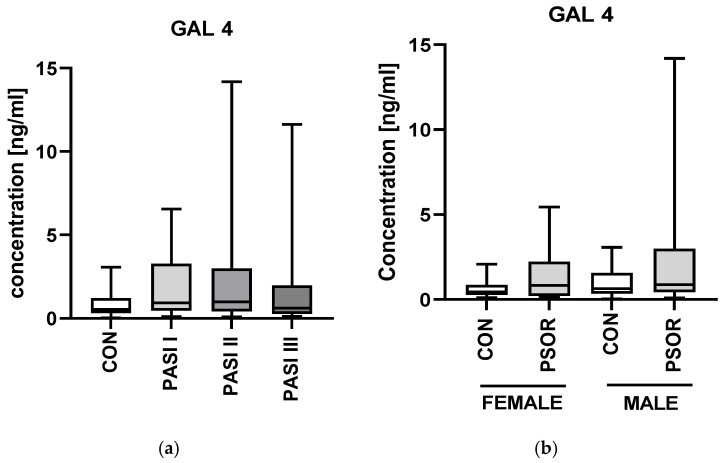

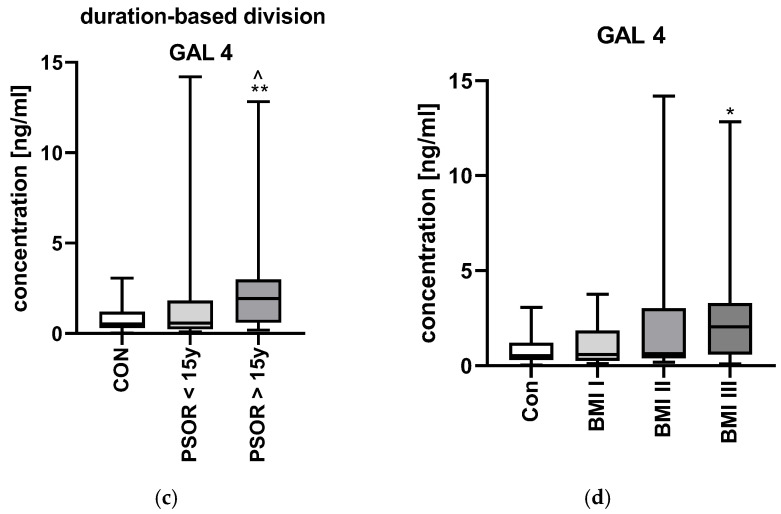
Plasma gal-4 concentration in patients divided regarding PASI (**a**), gender (**b**), psoriasis duration (**c**), and BMI (**d**) compared to controls. ^** means a statistically significant difference compared to the control group with *p* < 0.01, * means a statistically significant difference compared to the control group with *p* < 0.05 (CON *n* = 30, PSOR *n* = 60).

Regarding correlations with laboratory parameters, we noted a strong significant positive correlation between gal-4 and AST activity (R = 0.42; *p* < 0.0001) and an almost significant relation with ALT activity (R = 0.25, *p* = 0.05) (Figure 2b). There were no important differences in gal-4 concentrations between the PASI subgroups or gender division (NS) (Figure 3a,b).

Plasma gal-4 concentration was significantly higher in patients with longer than 15 years of lasting disease (*p* < 0.01) (Figure 3c). Further, the protein was the highest in psoriatics with obesity (*p* < 0.05) (Figure 3d).

### 2.2. CLC/Galectin10

**Figure 4:**
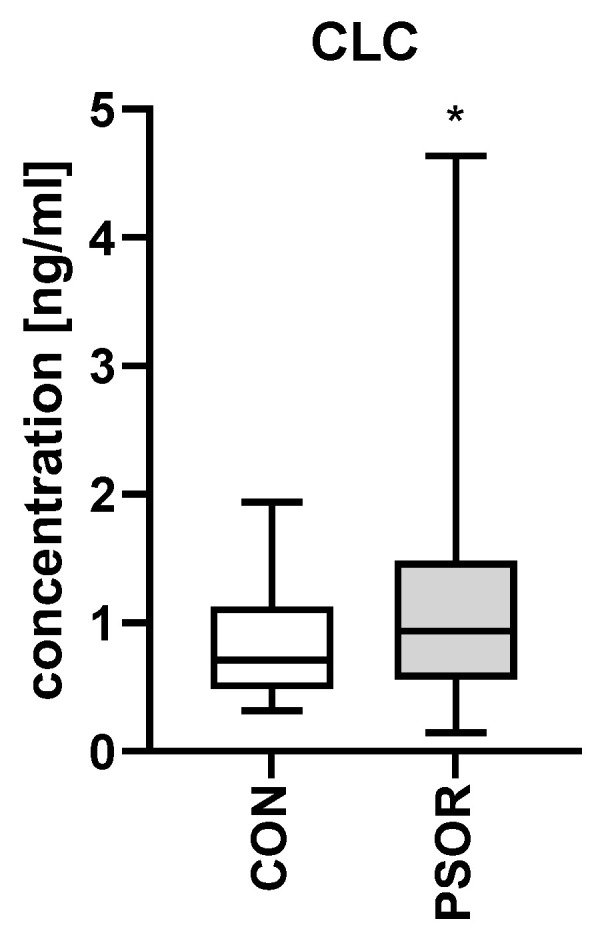
Plasma CLC/gal-10 concentration in patients compared to controls. * means a statistical significance with *p* < 0.05, (CON n = 30, PSOR n = 60).

The plasma concentration of Charcot-Leyden crystal protein/galectin-10 was significantly higher in the study group compared to the controls (*p* < 0.05) (Figure 4).

**Figure 5:**
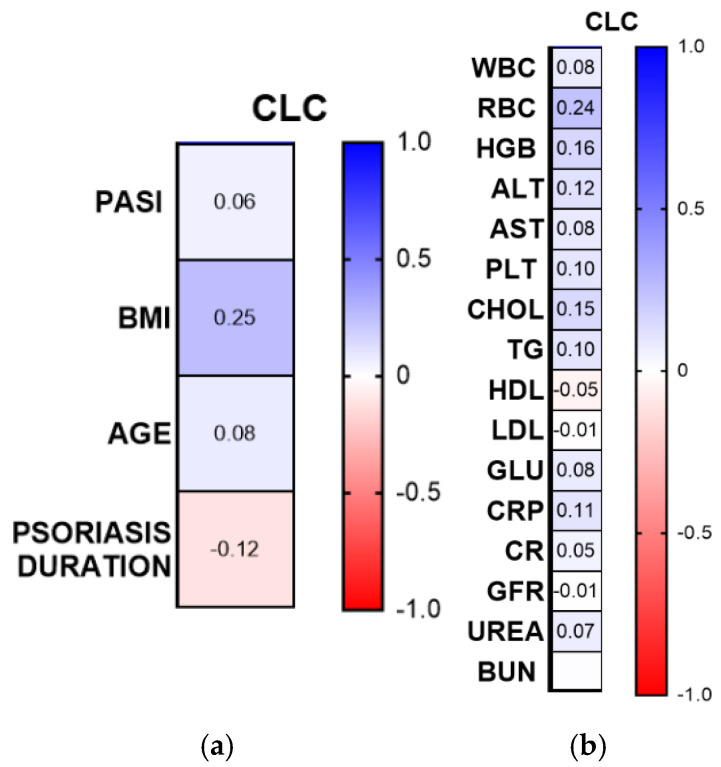
Correlations between plasma CLC/galectin-10 concentrations and clinical parameters (**a**) or laboratory indices (**b**). TGs, triglycerides; ALT, alanine aminotransferase; AST, asparagine aminotransferase; GLU, fasting glucose; Chol, total cholesterol; HDL, high-density lipoprotein; LDL, low-density lipoprotein; RBC, red blood cells; WBC, white blood cells; PLT, platelets; HGB, hemoglobin; CR, creatinine.

CLC/gal-10 did not correlate with PASI score nor age or disease duration; however, almost significantly positively correlated with BMI (R = 0.25, *p* = 0.054) (Figure 5a).

In relation to laboratory indices, no significance was noted (Figure 5b).

**Figure 6:**
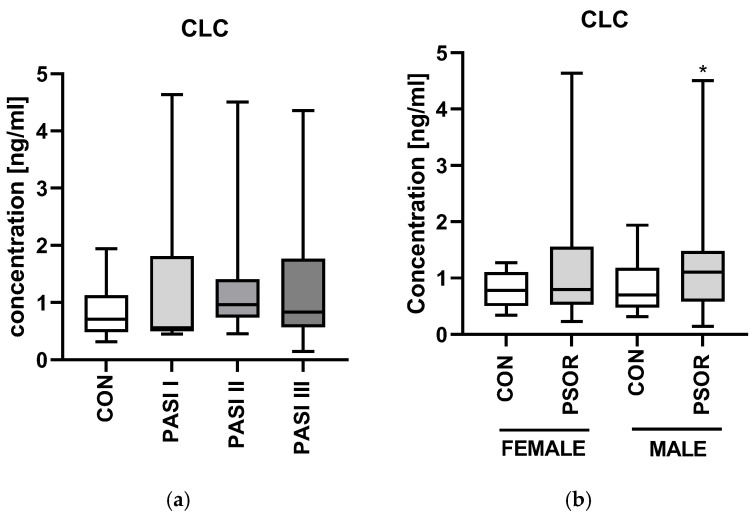

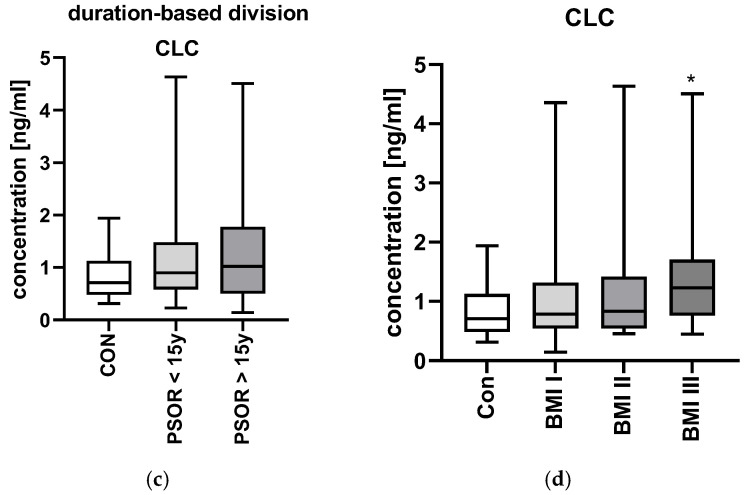
Plasma CLC/gal-10 concentration in patients divided regarding PASI (**a**), gender (**b**), psoriasis duration (**c**), and BMI (**d**) compared to controls (CON n = 30, PSOR n = 60). * means a statistically significant difference compared to the control group with *p* < 0.05.

There were no important differences in CLC/gal-10 concentrations between the PASI and disease-duration subgroups and healthy subjects (NS) (Figure 6a,c).

Plasma CLC/gal-10 level was significantly higher in psoriatic men and was the highest in obese psoriatics than in the controls (*p* < 0.05) (Figure 6b), (CON n = 30, PSOR n = 60).

## 3. Discussion

Galectins have emerged as a focus of increasing scientific interest, as valid regulators of the immune system, whose dysregulation is inextricably linked with the pathogenesis of psoriasis. Nevertheless, given the considerable heterogeneity within this protein family, many aspects of their role remain to be elucidated. To date, galectin-3 is the most extensively investigated member in the context of psoriasis, for which elevated serum levels have been consistently reported, including our own research [11]. Additionally, we have demonstrated a potential role of galectin-2 and -12 in this dermatosis [12]. Other galectins have been identified in various skin cells, T lymphocytes, macrophages, adipocytes, vascular endothelial cells and have been linked to certain skin conditions, including atopic dermatitis, contact dermatitis, psoriasis, skin cancers and wound healing [24,25].

To the best of our knowledge, we are the first to demonstrate significantly higher plasma concentrations of Charcot–Leyden crystal protein and galectin-4 in patients with psoriasis compared to individuals without the dermatosis. The scarcity of research on the role of both galectins in psoriasis imposes significant limitations on the discussion, as we can only refer to studies concerning other psoriasis-related disorders.

### 3.1. Galectin-4

Patients with psoriasis are at a higher risk of developing various autoimmune disorders, including IBD [26,27]. Yu T. et al. demonstrated that the serum level of galectin-4 was numerically higher in IBD patients than in controls, although the difference was insignificant [16]. Gobbi R. et al. showed the highest expression of gal-4 among the studied galectins in healthy colonic biopsies. Interestingly, they observed a decrease in gal-4 mRNA levels in inflamed intestinal areas of patients with active IBD. However, after clinical remission of IBD, tissue expression of gal-4 returned to normal levels, highlighting its potential use in monitoring disease activity [28]. Considering these two pieces of evidence, it can be concluded that the function of gal-4 in IBD appears to be limited to local mucosal mechanisms. In psoriasis, gal-4 may have a systemic role that needs to be elucidated. Hokama A. et al. demonstrated in mice that gal-4 worsened intestinal inflammation by stimulating CD4+ T cells to produce IL-6 [29]. In contrast, Paclik D. et al. presented evidence that gal-4 reduced inflammation by inducing T cell apoptosis and decreasing the release of proinflammatory cytokines (IL-6, IL-8, IL-10 and IL-17) [30]. The findings from both studies suggest that the protein can play a complex role in intestinal inflammation, exerting both anti-inflammatory and proinflammatory effects. Based on the data and our own findings, it can be hypothesized that gal-4 might be a novel biomarker for psoriasis and potentially contributes to the excessive inflammatory response in this condition.

Gal-4 has been associated with the development of several cardiometabolic diseases (CMDs). Obesity is one of the most common comorbidities of psoriasis, with multifactorial interrelations [31,32]. Korduner J. et al. suggested that elevated gal-4 levels are associated with a higher probability of hospitalizations of obese patients with diabetes [33]. That indicates gal-4’s potential involvement in diabetes-related complications. Additionally, Dieden A. et al. arrived at similar conclusions that increased levels of gal-4 are associated with obesity, diabetes, particularly in individuals with heart failure [34]. Molvin J. et al. identified gal-4 as a biomarker of both incident and prevalent type 2 diabetes [35]. Moreover, a correlation between gal-4 and increased risk of heart failure, future myocardial infarction, cardiovascular and all-cause mortality was established [36]. The close relation of these disorders to psoriasis, along with our outcomes, undeniably points to a significant role of gal-4 in this dermatosis and its complications. Gal-4 facilitates the transport of dipeptidyl peptidase-4 (DPP-4) to the intestinal epithelium, leading to decreased incretin activity and contributing to impaired insulin secretion, prediabetes and its cardiometabolic complications. Mohanty I.R. et al. observed significant positive correlations between DPP-4 levels and hemoglobin A1c (HbA1c), lipids and systolic blood pressure in rats [37]. Taken together, these findings suggest gal-4 may represent a molecular link between metabolic dysregulation and the chronic inflammation of psoriasis. Moving forward, patients with severe psoriasis are at increased risk of developing stroke [38]. Interestingly, Jujic A. et al. demonstrated that gal-4 increases in response to ischemic stroke [39]. The authors proposed that it may play a role in post-stroke inflammation through monocyte activation and vascular remodeling, processes also observed in psoriasis. That indicates a newer shared pathogenic pathway between stroke and psoriasis mediated by gal-4.

In our research, galectin 4 was significantly positively related to both BMI and psoriasis duration. Taken together, along with the data on obesity, gal-4 presumably acts as a molecular mediator connecting metabolic conditions with psoriasis and its chronic inflammation intensifies with the duration of the disease. Given the established connection between psoriasis and metabolic syndrome, as well as the frequent coexistence of obesity and dyslipidemia in psoriatic patients, the observed association between gal-4 levels and BMI highlights its potential role as a molecular mediator bridging metabolic and inflammatory pathways. Moreover, the positive relationship between gal-4 and disease duration suggests that its expression may increase as the inflammatory process becomes more chronic. Such chronic activation may influence keratinocyte activity and psoriatic inflammation.

Proinflammatory cytokines, which are elevated in psoriasis, may contribute to the development and progression of NAFLD [40,41]. Higher PASI scores, elevated BMI and abnormal liver enzyme levels have been associated with liver steatosis in patients with psoriasis [42]. In individuals with psoriasis, it is advisable to monitor liver enzymes like AST and ALT, since their elevated levels may indicate coexisting liver dysfunction, such as NAFLD; however, enzyme activity does not always correlate with the severity of liver damage [43]. Strong correlations between gal-4 levels and transaminases activity presented may indicate a potential link between the proteins and liver dysregulation in psoriatics. These findings should stimulate further research in this area.

### 3.2. Galectin-10/CLC

Galectin-10 has never been studied in psoriasis. We observed a markedly elevated level of plasma gal-10 compared to healthy subjects. It is regarded as an indicator of eosinophil-driven inflammatory responses. Noh S. et al. observed that its elevated levels are present in IL-22-expressing T cells from the peripheral blood and affected skin of individuals with AD. Moreover, these levels correlated positively with AD severity [23]. The authors suggested that gal-10 contributes to the excessive proliferation of keratinocytes, commonly observed in chronic lesions of AD. This potential link between gal-10 and keratinocytes may also be relevant in psoriasis, in which keratinocyte hyperproliferation is a key feature. Galectin-10 may also be implicated in the pathogenesis of another dermatological condition, bullous pemphigoid. Takahiko X. et al. discovered elevated levels of gal-10 and some metalloproteinases (MMPs) in the blister fluid of bullous pemphigoid patients. Gal-10 increases the expression of MMPs in human keratinocytes and fibroblasts, contributing to damage of the dermo-epidermal junction. This underscores the plausibility that gal-10 may participate in structural remodeling or barrier disruption in psoriatic lesions [44]. Additionally, serum gal-10 concentration was elevated in patients with eosinophilic granulomatosis with polyangiitis [45].

Interestingly, gal-10 has been found to be expressed in the placental tissue of women with gestational diabetes mellitus (GDM) [46]. This suggests that gal-10 may play a role in the inflammatory mechanisms underlying the pathogenesis of this metabolic disease. The study conducted by Unverdorben et al. demonstrated that gal-10 expression was markedly reduced in placentas from women experiencing spontaneous and recurrent miscarriages compared to healthy controls. This finding bolsters the plausibility that gal-10 is not only a circulating biomarker but might also act at the tissue level. The decrease in gal-10 in failing trophoblast could mirror possible dysregulation in psoriatic lesional skin, where aberrant expression might contribute to barrier disruption or abnormal remodeling [47].

Our findings revealed an almost significant positive correlation between gal-10 and BMI, suggesting its potential involvement in obesity, a component of metabolic syndrome, a major comorbid condition associated with psoriasis. This observation supports the concept that metabolic dysregulation and chronic inflammation are tightly interconnected in psoriatic disease.

## 4. Materials and Methods

The study enrolled 60 patients (21 women and 39 men) with exacerbation of plaque psoriasis, with a mean age of 49 ± 2.3 years old. Patients were compared to 30 sex-, age- and BMI-matched healthy volunteers without skin diseases. All participants signed an informed consent before initiation. The participants fulfilled the exclusion criteria: age under 18 years old, pregnancy, types of psoriasis other than plaque, dietary restrictions, intake of oral medications at least 3 months prior to the study, infectious or autoimmune diseases (other than psoriasis), and current or 5 years in the past history of malignant neoplasms. The severity of psoriatic skin lesions was evaluated by the Psoriasis Area and Severity Index (PASI) by the same dermatologist in all patients.

The study group was divided into three subgroups depending on psoriasis severity: PASI I (PASI < 10) meant mild form, PASI II (PASI 10-20)—moderate psoriasis, and PASI III (PASI > 20)—severe course. Patients were also divided regarding disease duration: more or less than 15 years. Body mass index (BMI) was calculated as weight/height2. The study group was divided in relation to BMI; group 0 meant the healthy controls, group 1—normal-weight (18.5–24.9) patients with psoriasis, group 2—indicated overweight psoriatics (BMI 25–29.9), group 3 was for obesity (BMI > 30).

Levels of highly sensitive C-reactive protein (hs-CRP), complete blood count (CBC), serum glucose, total cholesterol (Total Chol), HDL (high-density lipoprotein), LDL (low-density lipoprotein), triglycerides (TGs), and indicators of kidney and liver functions were measured before inclusion. The study was approved by the Bioethics Committee of the Medical University of Bialystok (number: APK.002.19.2020) and was conducted in accordance with the principles of the Helsinki Declaration [Helsinki].

### 4.1. Plasma Collection

Fasting blood samples were taken using vacuum tubes. They were centrifuged for 10 min at 2000× *g*. The plasma was stored at −80 °C until further assessment. Laboratory indices were measured using routine techniques. Gal-4,-10 plasma concentrations were measured with an enzyme-linked immunosorbent assay (ELISA) provided by Cloud Clone^®^ (Houston, TX, USA; SEA304Hu, SEC387Hu). Optical density was read at a wavelength of 450 nm. The concentrations were evaluated by interpolation from calibration curves with standard samples provided by the manufacturer. All laboratory tests were performed by the same person in standardized laboratory settings.

### 4.2. Statistical Analysis

Shapiro–Wilk’s W test of normality was used for data distribution analysis. The normally distributed data were analyzed using the Student’s *t*-test or one-way analysis of variance (ANOVA) and shown as mean ± SD. The non-Gaussian data were presented as median (full range) with the use of the non-parametric Mann–Whitney test or Kruskal–Wallis test. The links between the examined parameters were assessed with Spearman’s rank test. Statistical analysis was performed using GraphPad Prism 9.4 software. The differences were deemed statistically significant when *p* < 0.05.

## 5. Conclusions

In this study, we evaluated plasma levels of galectin-4 and galectin-10 in patients with psoriasis. To our knowledge, we are the first to demonstrate their potential involvement in pathogenesis and the course of this dermatosis. Our findings revealed that gal-4 and gal-10 were significantly higher in psoriatic patients compared to healthy controls. Gal-4 could be considered a novel biomarker in psoriasis, reflecting chronicity of systemic inflammation. As gal-4 has been linked to CMDs, it may be involved in metabolic or cardiac complications in psoriasis. Its association with liver enzyme activity suggests a potential role in monitoring liver function in psoriasis or as a predictor of liver disorders in psoriatics. Regarding both chronic inflammatory skin disorders, psoriasis and AD, gal-10 could be linked with their underlying immune dysregulation and keratinocyte hyperproliferation. Notably, both galectins may be considered involved in the interplay between obesity and psoriasis.

### Limitations

The primary limitation of the study is the limited heterogeneity of the participants, with a majority of male individuals from the same geographic area and ethnic background. Only patients with plaque psoriasis were included; the results may not be relevant for other psoriasis subtypes. These findings should be interpreted as preliminary and hypothetical, given the current lack of comparable studies for validation.

## Figures and Tables

**Table 1:** Basic characteristics of the participants.

**Parameter**	**Controls (*n* = 30)**	**Psoriasis (n = 60)**
Sex (M/F)	20/10	39/21 NS
Age [years]	47 ± 2.5	49 ± 2.3 NS
Height [cm]	1.7 ± 0.01	1.7 ± 0.01 NS
Weight [kg]	78 ± 3	84 ± 2 NS
BMI	25.7 ± 0.77	27.6 ± 0.8 NS

NS, non-significant.

## Data Availability

Data is contained within the article.

## Abbreviations

The following abbreviations are used in this manuscript:

TGs	Triglycerides
ALT	Alanine aminotransferase
AST	Asparagine aminotransferase
GLU	Fasting glucose
Chol	Total cholesterol
HDL	High-density lipoprotein
LDL	Low-density lipoprotein
RBC	Red blood cells
WBC	White blood cells
PLT	Platelets
HGB	Hemoglobin
CR	Creatinine
